# Carbon Based Polymeric Nanocomposites for Dye Adsorption: Synthesis, Characterization, and Application

**DOI:** 10.3390/polym13030419

**Published:** 2021-01-28

**Authors:** Moonis Ali Khan, Ramendhirran Govindasamy, Akil Ahmad, Masoom Raza Siddiqui, Shareefa Ahmed Alshareef, Afnan Ali Hussain Hakami, Mohd Rafatullah

**Affiliations:** 1 Chemistry Department, College of Science, King Saud University, Riyadh 11451, Saudi Arabia; mokhan@ksu.edu.sa (M.A.K.); mrsiddiqui@ksu.edu.sa (M.R.S.); 438203872@student.ksu.edu.sa (S.A.A.); 437203979@student.ksu.edu.sa (A.A.H.H.); 2 School of Industrial Technology, Universiti Sains Malaysia, Penang 11800, Malaysia; ramendhirran@student.usm.my (R.G.); akilahmad@usm.my (A.A.)

**Keywords:** graphene oxide, polyaniline, nanocomposite, brilliant green, adsorption modeling

## Abstract

Agglomeration and restacking can reduce graphene oxide (GO) activity in a wide range of applications. Herein, GO was synthesized by a modified Hummer’s method. To minimize restacking and agglomeration, in situ chemical oxidation polymerization was carried out to embed polyaniline (PANI) chains at the edges of GO sheets, to obtain GO-PANI nanocomposite. The GO-PANI was tested for the adsorptive removal of brilliant green (BG) from an aqueous solution through batch mode studies. Infrared (FT-IR) analysis revealed the dominance of hydroxyl and carboxylic functionalities over the GO-PANI surface. Solution pH-dependent BG uptake was observed, with maximum adsorption at pH 7, and attaining equilibrium in 30 min. The adsorption of BG onto GO-PANI was fit to the Langmuir isotherm, and pseudo-second-order kinetic models, with a maximum monolayer adsorption capacity (q_m_) of 142.8 mg/g. An endothermic adsorption process was observed. Mechanistically, π-π stacking interaction and electrostatic interaction played a critical role during BG adsorption on GO-PANI.

## 1. Introduction

Natural and human activities are deteriorating surface and sub-surface water quality. Domestic, sewage, industrial, and agricultural waste effluents are of major concern in this regard. These effluents are enriched with heavy metal (cadmium, lead, nickel), inorganic (nitrate, phosphate, sulfate), and organic (phenols, dyes, pharmaceuticals) contaminants. Among them, dyes have a wide range of applicability in the textile, paint, food, cosmetic, and pharmaceutical industries [1,2,3]. Approximately 10–15% of dyes are lost during their processing operations. Most dyes, and especially azo-dyes, are toxic to biota [4,5]. Excessive dye concentrations in aquatic system can cause eutrophication, which is harmful for both aquatic plants and animals.

Brilliant green (BG), synonymously called emerald green, is a cationic triarylmethane dye, with the chemical formula: C_27_H_33_N_2_·HO_4_S; molar mass: 482.64 g/mol; melting point: 210 °C; and IUPAC name: 4-{[4-(diethylamino) phenyl] (phenyl) methylidene}-N, N-diethylcyclohexa-2,5-dien-1-iminium]. Physically, it is shiny with small golden crystals. It is water and alcohol soluble, and used as an antiseptic agent against Gram-positive microorganisms. The industrial applications of BG include the coloring of wool and silk. Eye burns, skin irritation, coughing, nausea, vomiting, and diarrhea are some of the reported health issues triggered by consuming BG contaminated water [6]. Thus, it is essential to remove/minimize BG from waste effluents before their discharge into water bodies. The complex structure of BG restricts conventional water treatment approaches (such as biological treatment and chemical precipitation) for its abatement. Catalytic degradation, flocculation, aerobic or anaerobic digestion, coagulation, reverse osmosis, and adsorption are some of the efficient and highly acclaimed water remediation techniques [7,8,9,10,11]. Adsorption, owing to high efficiency, operational ease, and low cost has the upper hand among them [12].

Since the last decade, nano-sized adsorbents such as carbon nanotubes, nanofibers, polymer-based nanomaterials, metal oxide nanomaterials, etc. have been used in water purification and desalination [13,14,15]. Among them, graphene oxide (GO), a two-dimensional carbon framework with honeycomb-like structure, has gained considerable attention for water treatment applications in recent times. GO is enriched with hydrophilic functional groups such as hydroxyl and carboxyl which have excellent potential for adsorbing cationic dyes [16,17]. However, strong π-π stacking interactions between graphene nanosheets can lead to serious agglomeration and restacking, which reduces GO adsorption activity [18]. Hence, for sustainable adsorption performance, efforts have been made on the development of GO composites with good hydrophilicity and dispersion. For example, through in situ polymerization using aniline in the presence of GO [19]. Chemical alteration of the surface functional groups on GO (which act as chemical binding sites), may enhance their adsorption and dispersion capacity. Lately, polyaniline (PANI), because of its cost-effectiveness and ecologically appealing properties, has gained attention in water treatment operations. PANI possesses abundant amine and imine groups in its polymer chains, which makes it a promising material for compositing with GO.

In this study, PANI chains were embedded at the edge of GO sheets by in situ chemical oxidation polymerization to obtained a GO-PANI nanocomposite. The GO-PANI nanocomposite was tested for the adsorption of cationic brilliant green (BG) dye from water. The effects of experimental parameters such as pH, contact time, and initial BG concentration were studied to optimize the adsorption process. An interaction mechanism of BG dye onto GO-PANI was proposed. The results showed that GO-PANI is an effective adsorbent for the removal of cationic BG dyes from aqueous solutions.

## 2. Materials and Methods

### 2.1. Materials and Chemicals

Natural graphite flakes, aniline (C_6_H_5_NH_2_, 99%), potassium permanganate (KMnO_4_), hydrogen peroxide (H_2_O_2_), methanol (CH_3_OH), and hydrochloric acid (HCl) were procured from Sigma-Aldrich, Germany. Sulfuric acid (H_2_SO_4_), sodium nitrate (NaNO_3_), ammonium persulfate (APS: (NH_4_)_2_S_2_O_8_), and nitric acid (HNO_3_) were purchased from Fisher Scientific Ltd. Brilliant green (BG: C_27_H_34_N_2_O_4_S), used as a model adsorbate was purchased from Sigma-Aldrich, Germany, analytical regent (A.R) grade chemicals and regents were used during the study, without further purification. Deionized (D.I) water was used throughout the experiments.

### 2.2. Synthesis of Graphene Oxide (GO)

Hummer’s method, with some modifications, was used to synthesize GO [20]. Briefly, in a 500-mL round bottom flask, 12 g of NaNO_3_ was mixed for 2 h in 200 mL concentrated H_2_SO_4_ under continuous magnetic stirring at 80 rpm. Graphite flasks (10 g) under continuous stirring for 10 min were suspended in a mixture to obtain a dark color suspension. Thereafter, the suspension was kept in an ice bath and 18 g of KMnO_4_ was slowly added to the dark-colored suspension. The suspension was continuously stirred for 36 h at 50 °C to obtain a reddish-brown viscous mixture. Next, the mixture was cooled to room temperature, and 400 mL of cold D.I water containing 10 mL of H_2_O_2_ (50%, *v/v*) was poured into the mixture. Subsequently, the suspension was centrifuged and washed consecutively with HCl, D.I water, and finally with methanol a few times until the solution pH reached 6. The solid product was collected and put in an oven at 50 °C for 24 h. Thereafter, the solid powder product was dispersed in 1000 mL of D.I water, and sonicated (Cole Parmer-8891) for an hour. Finally, the product was filtered, and again dried in an over at 50 °C for 24 h.

### 2.3. Fabrication of Graphene Oxide (GO)/Polyaniline (PANI) Nanocomposites

The GO/PANI nanocomposite was synthesized through in situ polymerization on aniline in the presence of GO. Then, 50 mL of H_2_SO_4_ solution of 0.2 M concentration was prepared and equally divided into two parts, of 25 mL each. In first part, 0.2 M aniline and 5 gm GO were added, and ultrasonically mixed for 30 min. Thereafter, the mixture was continuously stirred for about 5 h at 5 °C for a better yield. In second part, 0.2 M APS was mixed with 25 mL 0.2 M H_2_SO_4_. Thereafter, the 0.2 M APS mixture was added drop by drop to the first mixture under continuous stirring, turning the mixture a greenish tint and then to violet. A 6 to 7 h aged mixture was turned into a black precipitate. Finally, the precipitate was consecutively washed with ethanol and D.I water, and dried for 24 h in an oven at 80 °C.

### 2.4. Characterization of GO/PANI Nanocomposite

Fourier transform infrared absorption (FT–IR: Perkin Elmer 2000, Waltham, MA, USA) spectra of pristine and BG saturated GO/PANI nanocomposite were recorded using a KBr pellet technique. Powder X-ray diffraction (PXRD: Bruker D8 Advance, Billerica, MA, USA) patterns of GO/PANI nanocomposite were recorded with Cu Kα radiation in a scanning range of 5–60 (2 theta) with a scan rate of 12/min. The thermal stability of the GO/PANI nanocomposite was tested by thermogravimetric analysis (TGA: Mettler Toledo, Stockholm, Sweden) and differential scanning calorimetry (DSC: Perkin Elmer DSC 6, Waltham, MA, USA) by pursuing N_2_ gas as a carrier at a flow rate of 100 mL/min within a temperature range 50–800 °C for TGA, and 50–600 °C for DSC, respectively, and at a heating rate of 10 °C/min. The morphology of the GO/PANI nanocomposite was observed by scanning electronic micrograph (SEM: Quanta FEG 650, thermofisher, Beverly, MA, USA), and transmission electron microscopy (TEM: ZEISS LIBRA 120 field emission electron microscope, Pleasanton, CA, USA) operated at 200 keV.

### 2.5. Dye Adsorption and Quantification

Batch scale BG adsorption studies were carried in a series of 100 mL Erlenmeyer flasks. Then, 50 mL BG solutions of varied initial concentrations (*C_o_*) in the range: 25–250 mg/L were equilibrated for 8 h with 0.05 GO/PANI nanocomposite dose (m) over a water bath shaker at 200 rpm and at room temperature. The effect of pH on BG adsorption was tested for the adsorbate pH range: 1–10. The pH of the solutions was adjusted by using suitable buffer solutions. Furthermore, to optimize the equilibrium time for the removal of BG from aqueous solutions, adsorption studies were carried out for contact time (t) in the range: 5–180 min at *C_o_*: 200 mg/L.

Spectrophotometric (UV-Vis spectrophotometer, Shimadzu, Japan) analysis was carried out to quantitatively determine the residual BG at a maximum wave length, λ_max_: 625 nm. The respective BG adsorption capacity at equilibrium (*q_e_*), at any time t (*q_t_*), and percentage (%) adsorption were calculated as:

(1) $$q_{e}\left(\mathrm{mg}/g\right)=(C_{o}-C_{e})\times \frac{V}{m}\;\;\;$$

(2) $$q_{t}\left(\mathrm{mg}/g\right)=\left(C_{o}-C_{t}\right)\times \frac{V}{m}\;\;\;\;$$

(3) $$\%adsorption\;=\;\frac{C_{o}-C_{e}}{C_{o}}\times 100\;\;$$

where *C_o_*, *C_e_*, and *C_t_* are the initial, equilibrium, and any time t concentration of BG in the solution, respectively. The adsorbate volume and adsorbent amount were symbolized with *V* (L) and *m* (g), respectively.

## 3. Results and Discussion

### 3.1. Characterization of GO-PANI

**Figure 1:**
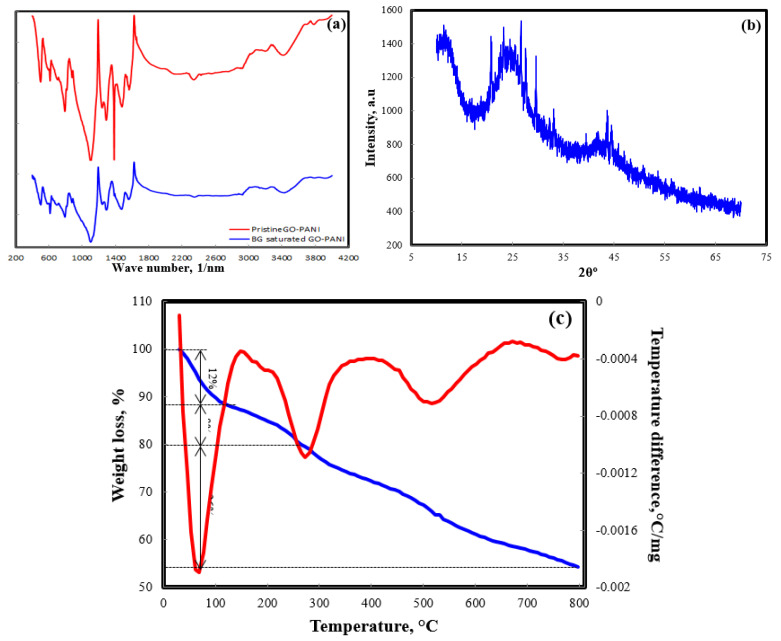
FT-IR (**a**), XRD (**b**), and TGA-DTA (**c**) plots of graphene oxide–polyaniline (GO-PANI).

Figure 1a illustrates the FT-IR spectra of pristine and BG saturated GO-PANI. In pristine GO-PANI spectrum, a broad band centered at 3500 cm^−^^1^, ascribed to O–H and N–H group stretching vibrations, was observed. A band at 2939 cm^−1^ was attributed to the bending vibration absorption peak of two substituted benzenes, in which C-H, i.e., carbon to hydrogen bond, is out-of-plane [21]. The bands in the GO-PANI spectrum at 1748, 1688, 1471, and 1234 cm^−1^ were attributed to C=O of the carboxyl group, aromatic C=C, carboxyl C–O, and the presence of carboxyl group O=C–O, respectively. A band at 1387 cm^−1^ was related to the C–O stretching. The bands at 1568, 1299, and 1097 cm^−1^ were attributed to C=C stretching of the quinonoid ring, C–N stretching of the secondary amine, and =N–H stretching, respectively The absorption band at 798 cm^−1^ was associated with the C–H out of plane bending vibration of the benzene ring. The observed FT-IR bands confirmed that PANI chains were embedded at the edge of the GO sheets. After BG adsorption over the GO-PANI nanocomposite, shifting and changes in band size were observed. The respective bands at 3500 and 1748 cm^−1^ were shifted to 3507 and 1755 cm^−1^, with a slight change in band size. This revealed the participation of hydroxyl and carboxylic groups during BG adsorption over GO-PANI.

The XRD patterns of GO-PANI nanocomposites showed a broad diffraction peak at 2θ = 25.5° (Figure 1b), this can be rationalized by the existence of oxygenated functional groups on the carbon sheets of GO-PANI nanocomposite [22]. A peak located at 2θ = 20.5° was ascribed to the (001) crystal plane of the layered structure of GO. The large interlayer distance was attributed to the formation of epoxy, hydroxyl, and carboxyl groups, which intensified the distance between the layers [23].

The thermal analysis (TGA-DTA) curve of GO-PANI showed a three step weight loss of the GO-PANI nanocomposite, illustrated in Figure 1c. A 12% weight loss during the first step occurred between 50 and 110 °C, which was associated to the evaporation of physically adsorbed water, and the disintegration of thermally labile oxygen-containing functional groups [24]. During the second stage, an 8% weight loss took place between 110 and 260 °C. This second stage weight loss was due to the loss of CO and CO_2_ from the breakdown of carbon oxidation, and oxygen containing functional groups, respectively. A major weight loss of 26% occurred between 260 and 800 °C, and was due to the release of dopant from the PANI surface (up to 460 °C), and the disintegration of PANI (above 460 °C) [25].

**Figure 2:**
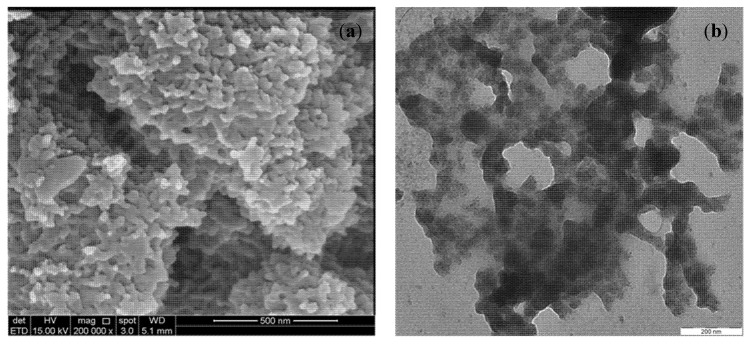
SEM (**a**) and TEM (**b**) images of GO-PANI.

Morphologically, the GO-PANI surface, due to the deposition of PANI nanofibers over GO nanosheets, was rough and uneven (Figure 2a). The edges extended in rod like particles, signifying that PANI was successfully grafted on the edges of the GO sheets. The spaces in the network comprised micro- and nano-pores, which provide enough pathways for the movement of dye ions [26]. The TEM image (Figure 2b) showed that PANI nanoparticles were well dispersed over the GO. Some wrinkled/folded structures were clearly seen, owing to structural defects, which might occur due to the presence of sp^3^ sites of oxygen-containing functionalities. These defects provided sufficient avenues to facilitate the diffusion of the BG dye ions [27].

### 3.2. pH Studies and BG Adsorption Mechanism

**Figure 3:**
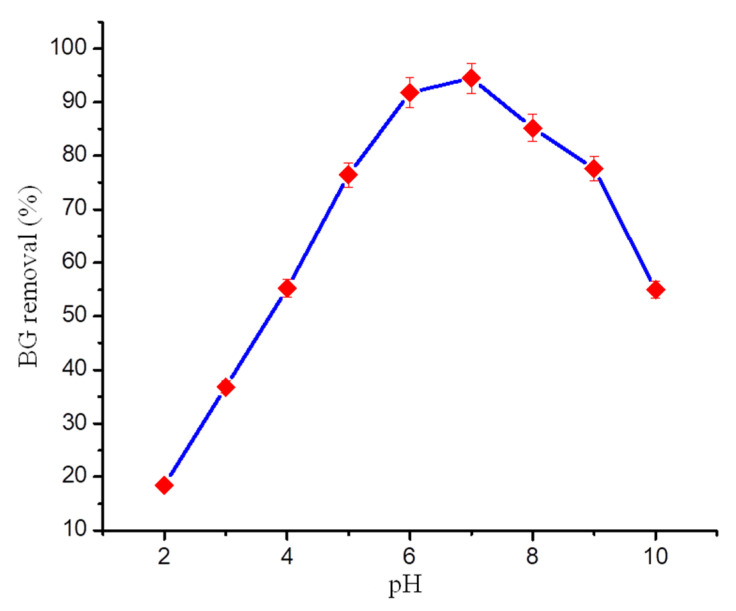
pH plot for BG adsorption on GO-PANI.

**Figure 4:**
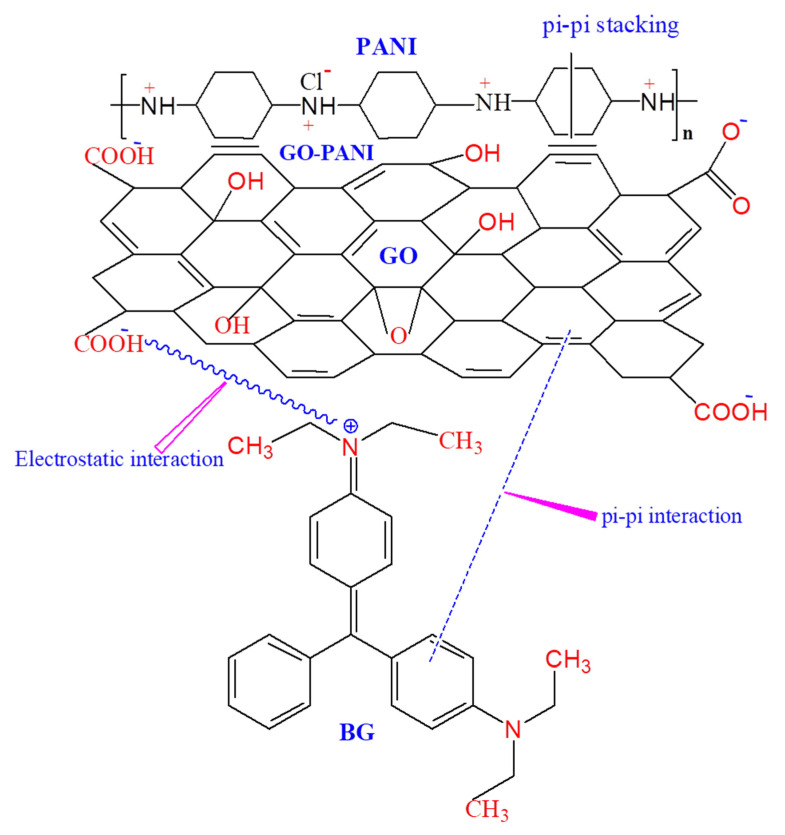
Brilliant green (BG) adsorption mechanism on GO-PANI.

The effect of pH on BG adsorption onto GO-PANI nanocomposite was examined in the pH range: 2–10, illustrated in Figure 3. A pH dependent BG uptake over GO-PANI nanocomposite was observed. This could be due to surface charge variation of adsorbent and dye molecule ionization with change in solution pH. The BG adsorption onto GO-PANI increased with the increase in solution pH, attaining optimum BG adsorption at pH 7. Furthermore, the increase in pH resulted in a decrease in BG adsorption. Similar adsorption behavior was observed elsewhere [28]. Mechanistically, the BG adsorption onto GO-PANI can be explained in terms of H-bonding, π-π interactions, Van der Waals force, and electrostatic attraction. The presence of a hydroxyl (–OH) and carboxylic (–COOH) group on the surface of GO-PANI (depicted during IR analysis), which contains negative charges in a basic medium, was basically responsible for increasing the chances of positively charged H_2_C–N^+^ of BG dyes being attracted. Therefore, the amino group of cationic BG dye (positively charged) and adsorbent surface (negatively charged) might be responsible for the electrostatic interaction (Figure 4). Moreover, the H-bonding may be considered the other option for interaction between the oxygen and nitrogen which are present on functional groups of GO-PANI and BG dye. The π-π stacking interactions between GO and BG molecules would also play a significant role during dye adsorption. Previously, researchers have studied the influence of pH on the BG adsorption efficiency, related to several types of adsorbents. However, the ideal pH for optimum BG adsorption was found the pH range: 6–8 [29,30]. Wang et al. [31] and Sharma [32] also stated that the hydroxyl (–OH) and carboxylic (–COOH) group and amine groups of BG were involved during the adsorption process.

### 3.3. Contact Time and BG Concentration Studies

**Figure 5:**
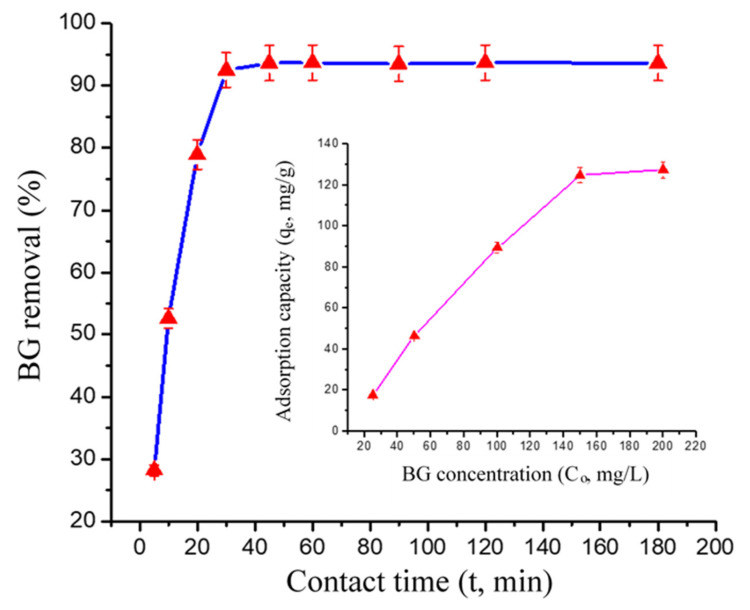
Effect of contact time on BG adsorption onto GO-PANI. Inset: Effect of concentration plot.

Figure 5 displays the contact time (t) and initial concentration (C_o_) (Figure 5, Inset) plots for BG adsorption onto GO-PANI in respective t and *C_o_* ranges: 5–180 min and 25–200 mg/L, at pH: 7 and temperature: 25 °C. In general, the BG adsorption on GO-PANI was directly proportional to t and *C_o_*. This could be due to the high inducing force of the concentration gradient with the increase in the *C_o_* of BG [33]. Based on the contact time curve, initially, BG adsorption was rapid, then progressively slowed down until it reached equilibrium. During the initial adsorption stage there were many unsaturated binding sites available over the GO-PANI surface, and as the BG adsorption proceeded these binding sites got saturated, and after some time, it was hard for BG to occupy the remaining vacant surface sites, because of repulsive forces which occur between bulk phases and the solute molecules. The maximum riddance of BG onto the GO-PANI composite materials was attained after 30 min, and after equilibrium thereon remained constant. Once equilibrium was achieved, the adsorption of BG was persistent with further time increment. Therefore, it was presumed that a longer proceeding of the reaction might not have further reactions to change the properties of the adsorbent. When the *C_o_* of BG increased from 25 to 200 mg/L, the adsorption capacity of GO-PANI escalated from 17.68 to 127.30 mg/g. It was noticed from the plot that at high concentration, the adsorption of BG onto GO-PANI was low, while at low concentrations the initial adsorption of dye was rapid, specifying a rapid surface reaction. Therefore, the concentration greatly influences the degree and rate of BG adsorption onto GO-PANI. Similarly to previous studies on BG onto poly (AN-co-VP)/zeolite composite [34], and nano hydroxyapatite/chitosan composite [35].

### 3.4. Kinetic Modeling

With the aim of determining the adsorption efficiency rate of BG onto the GO-PANI surface, the pseudo-first-order [36], and pseudo-second-order [37] kinetic models were investigated. Moreover, a Weber–-Morris diffusion model [38] was also studied.

The linear equation of the pseudo-first-order kinetic model can be articulated as follows:

(4) $$\log\;\left(q_{e}-\;q_{t}\right)=\;\log q_{e}-\;\frac{k_{1}}{2.303}\;\times \;t\;\;\;\;$$

where *q_e_* (mg/g), and *q_t_* (mg/g) are the amounts of BG adsorbed at equilibrium, and at any time (t) on GO-PANI, *k*_1_ is the pseudo-first-order rate constant (1/min), *t* is time (min).

**Figure 6:**
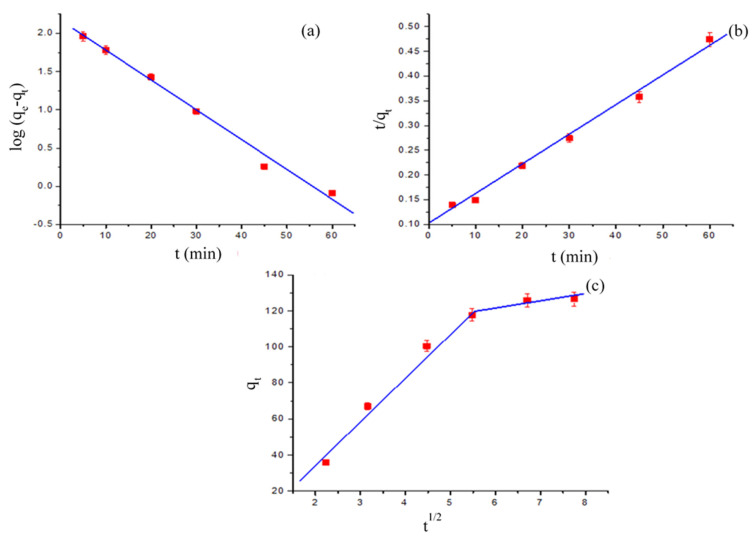
Pseudo-first-order kinetic plot (**a**), pseudo-second-order kinetic plot (**b**), and intraparticle diffusion plot (**c**) for BG adsorption on GO-PANI.

The numerical values of the kinetic parameters were computed from linear plots, log (*q_e_* − $q_{t}$) vs. t plot (Figure 6a) and tabulated (Table 1). From the results, the value of the correlation coefficient (R^2^) was very low, so it did not follow the pseudo-first-order kinetic model. Hence, the kinetic data was further examined by using the pseudo-second-order model, in linearized form, expressed as:

(5) $$\frac{t}{q_{t}}=\;\frac{1}{k_{2}q_{e}^{2}}+\;\frac{t}{q_{e}}\;\;\;\;$$

where $k_{2}$ is the pseudo-second-order rate constant (g/mg- min).

The values of kinetic parameters for pseudo-second-order were calculated from linear plots (*t*/*q_t_*) vs. *t* (Figure 6b), and the obtained results for kinetic parameters are given in Table 1. From the results, the R^2^ value for the pseudo-second-order was nearer to unity. To verify the kinetic model fitting to experimental data, Chi square (*X*^2^) test values were evaluated, expressed as:

(6) $$\;{Χ}^{2}=\sum^{\;}\frac{{\left(q_{e,exp.}-q_{e,cal.}\right)}^{2}}{q_{e,exp.}}\;$$

where *q_e,exp_*_._ and *q_e,cal_*_._ are the experimental and calculated adsorption capacities, respectively.

The *X*^2^ test results for the pseudo-first-order model showed a very high value, while for the pseudo-second-order model the value was very close to unity (Table 1), supporting the applicability of the pseudo-second-order model to kinetic data.

Thus, these results affirmed that the adsorption of BG onto GO-PANI followed the pseudo-second-order kinetics. Similar outcomes for BG adsorption were previously observed [35].

To examine the diffusion process of BG onto GO-PANI, a Weber–Morris diffusion model was studied. The linear equation for the Weber–Morris diffusion model is expressed as:

(7) $$q_{t}\;=\;k_{\mathrm{id}}\;t^{1/2}\;+\;C$$

where k_id_ = the diffusion rate constant (mg/g-min^1/2^).

The value of k_id_, C, and R^2^ was calculated from the gradient of plot *q_t_* vs. t^1/2^, as shown in Figure 6c, and the related data is presented in Table 1. The results revealed multi-linearity for BG adsorption onto GO-PANI, indicating that more than one rate determining step was involved during the adsorption process. The first linear section describes the external diffusion of BG onto the GO-PANI surface. Moreover, the second linear portion illustrates the diffusion (intra-particle) of BG, as a slowed process. On the other hand, the plot revealed that there are a few factors playing a significant role in the adsorption process, because of the multi-linearity correlation. The deviation in line where it does not pass through the origin, indicates that in the adsorption process film diffusion and intraparticle diffusion were involved [39].

### 3.5. Isotherm Modeling

In order to determine the most suitable model which represents the adsorption process, the affinity of adsorbent and adsorbate, as well as the surface property was studied with the help of isotherm models, viz., Freundlich [40], Langmuir [41] and Temkin [42]. The Langmuir model assumes that at homogeneous sites on the adsorbent surface the adsorption takes place, while in case of a monolayer the adsorbate is attached at the outer surface of the adsorbent. While, in the case of the Freundlich model, non-ideal and multilayer adsorption on heterogenous surface occurred, which was demonstrated by the heterogeneity factor *n*. The Temkin isotherm model assumes that along with the saturation of adsorption sites during adsorption, the heat of adsorption decreases linearly rather than exponentially. The Langmuir, Freundlich, and Temkin isotherm models in linearized forms are respectively expressed as:

(8) $$\frac{C_{e}}{q_{e}}=\frac{1}{bq_{m}}+\frac{1}{q_{m}}\times \;C_{e}\;\;\;\;\;\;$$

(9) $$ln\;q_{e}=ln\;k_{F}+\frac{1}{n}\times ln\;C_{e}\;\;$$

(10) $$q_{e}=Bln\;A+B\;ln\;C_{e}$$

where *C_e_* is the concentration at equilibrium (mg/L), *q_e_* is the amount of BG adsorbed on GO-PANI at equilibrium (mg/g), *q_m_* is the Langmuir constant related to adsorption efficiency (mg/g), b is the Langmuir constant related to adsorption energy(L/mg), *k_F_* is the Freundlich constant related to bonding energy ((mg/g)(L/mg)^1/n^), *n* is the deviation in adsorption from linearity, A is the binding constant at equilibrium corresponding to the maximum binding energy (L/g), and B is the Temkin constant related to the adsorption energy.

**Figure 7:**
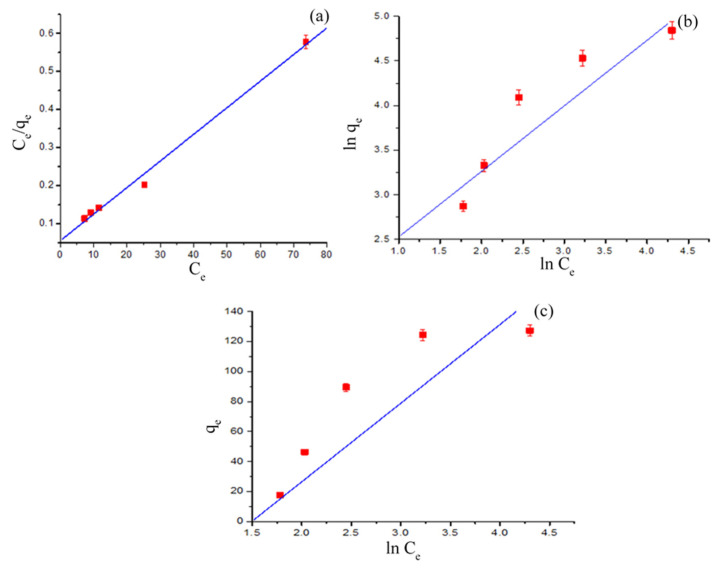
Langmuir (**a**), Freundlich (**b**), and Temkin (**c**) isotherm plots for BG adsorption on GO-PANI.

The isotherm plots are displayed in Figure 7, and Table 2 presents isotherm data. Among the studied models the R^2^ value for Langmuir was nearer to unity. This showed that compared to the Freundlich and Temkin models, the Langmuir model exhibited better fitting to adsorption data. Therefore, it was confirmed that no transmission of BG in the plane of surface and adsorption of BG onto the GO-PANI surface occurred homogeneously with constant energy. A comparison of GO-PANI with previously reported adsorbents for BG removal, along with the applied experimental conditions, is presented in Table 3 [6,34,35,43,44,45].

### 3.6. Thermodynamic Modeling

Thermodynamic parameters such as standard free energy change (ΔG°), entropy change (ΔS°), and enthalpy change (ΔH°) for BG adsorption on GO-PANI are calculated as:

(11) $$\Delta G{}^{\circ}=-RT\;ln\;K_{c}$$

(12) $$ln\;K_{c}=\frac{\Delta S{}^{\circ}}{R}-\frac{\Delta H{}^{\circ}}{R}\times \frac{1}{T}\;\;\;$$

where *T* is the absolute temperature (*K*), R is the universal gas constant (8.314 J/mol-K), and *K_c_* is the distribution coefficient.

**Figure 8:**
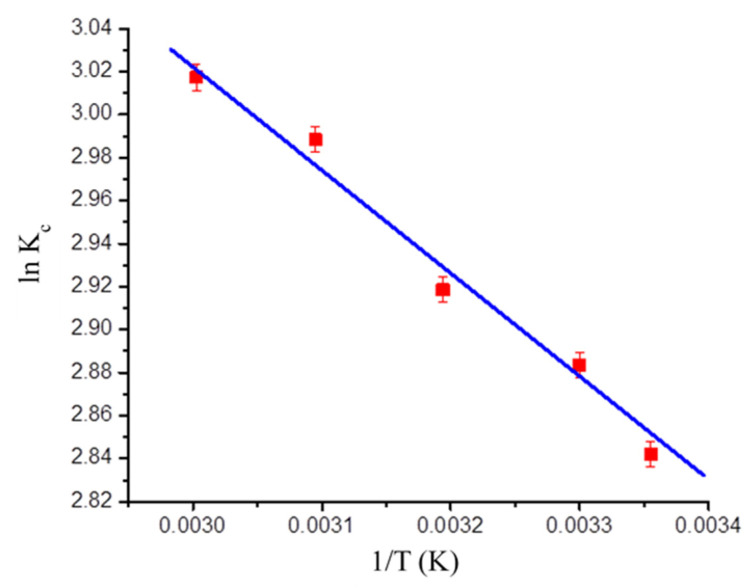
van’t Hoff plot for BG adsorption onto GO-PANI.

The ΔH° and ΔS° magnitudes were calculated from the slope and intercept of van’t Hoff’s plot (ln K_c_ vs. 1/T), illustrated in Figure 8. It can be seen from Table 4 that Δ*G*° values decreases from −7.0412 to −8.3535 kJ/mol when temperature increases from 298 to 333 K. The negative magnitude of Δ*G*° indicates the feasibility and spontaneity of the adsorption process. The positive value of Δ*H*° shows that BG adsorption on GO-PANI was endothermic in nature, while positive values of Δ*S*° confirmed the enhancement in disorderedness of the adsorbent particles during the adsorption of BG onto the GO-PANI composite at the solid–solution interface [46]. The energy was related to various physical forces, such as hydrogen bond forces (2–40 kJ/mol), hydrophobic bond forces (5 kJ/mol), van der Waals forces (4–10 kJ/mol), dipole bond forces (2–29 kJ/mol), coordination exchange (40 kJ/mol), and chemical forces (>60 kJ/mol) [47]. Experimental data shows that ΔH° was found to be 9.5569 kJ/mol. Hence, the results revealed that physical forces were involved during the adsorption of BG onto GO-PANI.

## 4. Conclusions

GO-PANI nanocomposite was successfully fabricated using an in situ chemical polymerization technique. Spectrometric analysis revealed that carboxylic and hydroxyl groups were abundantly present over the GO-PANI surface, and played a significant role during BG adsorption. A pH dependent BG uptake on GO-PANI, due to electrostatic and π-π interactions, was observed, while a maximum 92% BG removal was achieved at pH 7. The Langmuir isotherm model best fit the experimental data, which shows monolayer and homogeneous BG coverage over the GO-PANI surface during adsorption. Kinetic data was fit to the pseudo-second-order model. Thermodynamically, adsorption was spontaneous and endothermic, with ∆H° and ∆S° values of 9.557 kJ/mol and 37.654 kJ/mol-K, respectively. Thus, it can be concluded that the synthesized GO-PANI nanocomposite was cost-effective, efficient, and an alternate material for the elimination of BG from water and wastewater.

## Figures and Tables

**Table 1:** Kinetic modeling parameters for BG adsorption on GO-PANI.

Kinetic Models	Parameters				
Pseudo-first-order	*q_e,exp_*_._ (mg/g)	*q_e,cal_* (mg/g)	*k*_1_ (1/min)	R^2^	*Χ* ^2^
	127.3	33.56	0.9212	0.9607	69.02
Pseudo-second-order	*q_e,exp_*_._ (mg/g)	*q_e,cal_* (mg/g)	*k*_2_ (g/mg-min)	R^2^	*Χ* ^2^
	127.3	141.29	0.0043	0.9956	1.54
Intra-particle diffusion	k_id_ (mg/g·min^1/2^)		C	R^2^	
	16.49		13.67	0.8826	

**Table 2:** Isotherm modeling parameters for BG adsorption on GO-PANI.

Isotherm Models	Parameters		
Langmuir	*q_m_* (mg/g)	b (L/mg)	R^2^
	142.85	0.1263	0.9926
Freundlich	*k_F_* ((mg/g)(L/mg)^1/n^)	1/n	R^2^
	114.33	1.5151	0.6539
Temkin	A	B	R^2^
	6.8359	42.145	0.7916

**Table 3:** Comparison of adsorption capacities of various absorbents used for BG removal.

Adsorbent	Experimental Conditions	Adsorption Capacity (mg/g)	References
Red clay	C_o_: 20–100 mg/L; pH: 7; t: 240 min; T: 45 °C	125	[6]
Poly(AN-co-VP)/zeolite Composite	C_o_: 40.20 mg/L; m: 0.20 g/50 mL; t: 121.60 min	23.81	[34]
Nano-hydroxyapatite/chitosan composite	C_o_: 05–80 mg/L; t: 60 min; m: 0.9 g/L; pH: 7	49.1	[35]
Areca nut husk	m: 10 g/L; pH: 7.0; C_o_: 50–200 mg/L; T: 298 K; agitation speed: 200 rpm	18.21	[43]
NaOH treated saw dust	C_o_: 50–200 mg/L; pH: 2.9; t: 180 min; m: 4 g/L	58.48	[44]
Carboxy methyl cellulose/chitosan/graphene oxide	C_o_: 2–12 mg/L; pH: 7; t: 350 min	1.90	[45]
GO-PANI	C_o_: 25–200 mg/L; pH: 7; t: 30 min; T: 25 °C	142.85	This study

**Table 4:** Thermodynamic parameters for BG adsorption on GO-PANI.

Temperature (K)	Parameters			
	ΔG°(kJ/mol)	ΔH°(kJ/mol)	ΔS°(kJ/mol-K)	R^2^
298	−7.0412			
303	−7.2637			
313	−7.5954	9.5569	37.6541	0.9836
323	−8.0256			
333	−8.3535			

## Data Availability

The data presented in this study are available on request from the corresponding author.
